# Development of SNP and InDel markers by genome resequencing and transcriptome sequencing in radish (*Raphanus sativus* L.)

**DOI:** 10.1186/s12864-023-09528-6

**Published:** 2023-08-08

**Authors:** Yadong Li, Xiaobo Luo, Xiao Peng, Yueyue Jin, Huping Tan, Linjun Wu, Jingwei Li, Yun Pei, Xiuhong Xu, Wanping Zhang

**Affiliations:** 1 College of Agriculture, Guizhou University, Guiyang, 550003 China; 2 Institute of Vegetable Industry Technology Research, Guizhou University, Guiyang, 550003 China; 3 Guizhou Province Academy of Agricultural Sciences, Guizhou Institute of Biotechnology, Guiyang, 550003 China

**Keywords:** Radish, InDel, Genetic diversity, Population structure, Transcriptome, Flowering

## Abstract

**Background:**

Single nucleotide polymorphisms (SNPs) and insertions/deletions (InDels) are the most abundant genetic variations and widely distribute across the genomes in plant. Development of SNP and InDel markers is a valuable tool for genetics and genomic research in radish (*Raphanus sativus* L.).

**Results:**

In this study, a total of 366,679 single nucleotide polymorphisms (SNPs) and 97,973 insertion-deletion (InDel) markers were identified based on genome resequencing between ‘YZH’ and ‘XHT’. In all, 53,343 SNPs and 4,257 InDels were detected in two cultivars by transcriptome sequencing. Among the InDel variations, 85 genomic and 15 transcriptomic InDels were newly developed and validated PCR. The 100 polymorphic InDels markers generated 207 alleles among 200 Chinese radish germplasm, with an average 2.07 of the number of alleles (Na) and with an average 0.33 of the polymorphism information content (PIC). Population structure and phylogenetic relationship revealed that the radish cultivars from northern China were clustered together and the southwest China cultivars were clustered together. RNA-Seq analysis revealed that 11,003 differentially expressed genes (DEGs) were identified between the two cultivars, of which 5,020 were upregulated and 5,983 were downregulated. In total, 145 flowering time-related DGEs were detected, most of which were involved in flowering time integrator, circadian clock/photoperiod autonomous, and vernalization pathways. In flowering time-related DGEs region, 150 transcriptomic SNPs and 9 InDels were obtained.

**Conclusions:**

The large amount of SNPs and InDels identified in this study will provide a valuable marker resource for radish genetic and genomic studies. The SNPs and InDels within flowering time-related DGEs provide fundamental insight into for dissecting molecular mechanism of bolting and flowering in radish.

**Supplementary Information:**

The online version contains supplementary material available at 10.1186/s12864-023-09528-6.

## Introduction

Molecular marker is a valuable tool for genetics and breeding research in plant, such as fingerprinting genotypes, genetic map construction, QTL mapping, association analysis and marker-assisted selection (MAS) [1, 2]. In the past three decades, a large number of molecular markers have been successfully developed in plant, including restriction fragment length polymorphisms (RFLPs), random amplified polymorphic DNA (RAPDs), amplified fragment length polymorphisms (AFLPs), simple sequence repeats (SSRs), insertion/Deletions (InDels) and single-nucleotide polymorphisms (SNPs) [3, 4]. SSR and InDel are PCR-base markers with the advantage of bi-allelic, co-dominant, abundance and low-cost [5]. SNP and InDel are the most abundant genetic variations and widely distribute across the genomes in plant [6]. With the common characteristics of SSR, InDel markers have receive more and more attention. With the development of next-generation sequencing (NGS), InDel markers has extensively developed and applied in crop breeding [7, 8]. In soybean, a total of 17,613 InDels were detected in 56 soybean accessions and a genetic map with 300 InDel markers was constructed in 20 linkage groups [9]. The promoter of *Sl-ALMT9* with a 3 bp InDel was increased the expression levels of *Sl-ALMT9* and fruit malate contents in cultivate tomato [8]. In total, 47,558 InDels were identified between the two Cannabis accessions, 14 InDels were applied to perform the genetic structure analysis [7]. In mung bean, 129 InDel markers were developed and used to construct a genetic linkage map by the genome resequencing between two parents, the major effect QTL *qYSC4* for young stem color on chromosome 4 was narrowed in a 347 kb interval [5]. In all, 318 InDel markers were developed across the eight chromosomes by comparing the assembled genomic sequences of two *Medicago truncatula* varieties, *gibberellin 3-β-dioxygenase 1* gene for in the *dwarf* mutant *crs* were isolated [10].

Radish (*Raphanus sativus* L.) is an important root vegetable belonging to cruciferous family. A large number of InDel markers have been characterized and applied for genetic and genomic studies in radish [3, 11]. A total of 9,436 InDel were detected in three radish transcriptome and 40 InDel markers were used for genetic diversity analysis in 32 radish accessions [3]. In total, 99 EST-SSR and InDel markers were used to construct linkage groups and QTL mapping, QTL for late-bolting trait was located a 1.1-cM region between InDel520 and InDel535 [12]. Previous studies indicated that the *R* locus was identified between RsInDel4 and RsInDel11, and *RsMYB90* was defined as a candidate gene underlying the taproot skin color trait [13]. However, the number of InDel markers is far from sufficient for radish genetic studies.

The transition from the vegetative phase to the reproductive phase is the most important stages in the life cycle of flowering plants, which is control by multiple environmental signals and genetic pathway. Numerous studies have found that more than 300 genes associated with flowering time were mainly involved in six key pathways, including photoperiod, vernalization, ambient temperature, age, autonomy, and gibberellin pathways [14, 15]. In *Arabidopsis*, *FLOWERING LOCUS C* (*FLC*) as a repressor of flower plays crucial roles in the vernalization response [14]. *FLOWERING LOCUS T* (*FT*) in the photoperiod pathway as a central floral integrator was exhibited to delay flowering [16]. Bolting and flowering times as two important agronomic traits determine production and quality of radish. Previous studies showed that 142 bolting and flowering time related genes were acquired by transcriptome sequencing [17]. It was found that 218 homologs of *Arabidopsis* flowering-time genes were obtained in radish, of which 49 genes were identified as differentially expressed in two radish accessions [18]. A total of 254 flowering genes in *R. sativus* were characterized based on sequence similarities analysis [11]. Two QTLs each for bolting and flowering times were identified on chromosome R06 by QTL mapping [19]. A 1627-bp insertion near the 5′ end of the first intron of *RsFLC2* was associated with late-bolting trait in radish [12]. A large number of bolting and flowering genes have been reported in radish, no study on identify the genetic variation in flowering genes was performed in radish.

In this study, the SNP and InDel markers were developed by genome resequencing and transcriptome between early and late bolting cultivars. The InDel markers were developed and validated by PCR based markers. Population structure and clustering analysis were performed based on genotyping 200 radish cultivars with the developed InDel markers. The differential expressed genes (DEGs) of vegetative growth were identified between two cultivars. The homologs of flowering-time related gene (FTR) in radish were characterized. The transcriptomic SNP and InDel markers within flowering-time related genes were obtained. These results could provide an abundant marker for genetic and genomic studies in radish.

## Materials and methods

### Plant materials and DNA extraction

In total, 200 radish cultivars were collected from Institute of Vegetable Industry Technology Research in Guizhou University. The detail information of all cultivars are listed in Table S1. The seeds of two high-generation radish inbred lines ‘YZH’ (early-flowering time, 65d) and ‘XHT’ (late-flowering time, 185d) were planted in plastic pots and cultured in a chamber at 22 ℃ for 14 h light and 10 h dark. After 25 days, the young leaves of ‘YZH’ and ‘XHT’ were taken for three biological replicates for genome resequencing and transcriptome sequencing. Leaves of all radish cultivars were collected and immediately frozen in liquid nitrogen, and stored at -80 °C for further use. Total genomic DNA was extracted using the EasyPure Genomic DNA Kit (TransGen Biotech, Beijing, China) according to the manufacturer’s instructions. The DNA with a final concentration of 10 ng/ul were used to conduct PCR amplifications.

### Genome resequencing and identification of SNPs and InDels

The ‘YZH’ and ‘XHT’ in vegetative growth period was used to genome resequencing. Total genomic DNA was extracted and the quality of genomic DNA was quantified prior to library construction. According to manufacturer's recommendation, six sequencing libraries were generated using NEB Next® Ultra™ DNA Library Prep Kit for Illumina (NEB, USA). The DNA libraries were sequenced on Illumina platform to generate 150 bp paired-end reads. After removing low-quality reads, the clean reads were assigned to the radish reference genome using Burrows-Wheeler Aligner (BWA)-0.7.8 (parameter: mem -t 4 -k 32 -M) [20, 21]. The SNPs and InDels calling were performed using SAMtools-1.3.1 (mpileup -m 2 -F 0.002 -d 1000) [22]. The variants were filtered with the following criteria: (1) The depth of the variate position > 4; (2) The mapping quality > 20. The functional annotation of variants was carried out by ANNOVAR (Annotate Variation).

### RNA isolation and transcriptome sequencing

Total RNA were extracted from the young leaves of ‘YZH’ and ‘XHT’ in vegetative growth period (after 25 days after sowing) using a Plant RNA Mini Kit (Tiangen, Inc., China). To assess RNA integrity, the RNA Nano 6000 Assay Kit of the Bioanalyzer 2100 system (Agilent Technologies, CA, USA) were used [23]. A total amount of 1 µg RNA per sample was used as input material for the RNA sample preparations. Sequencing libraries were constructed using NEBNext® UltraTM RNA Library Prep Kit for Illumina® (NEB, USA). All libraries were sequenced on Illumina Novaseq platform.

### Analysis of differential expressed genes (DEGs) and function annotation

After removing adapter and low quality reads, clean reads were obtained. The clean reads were aligned to the radish reference genome (http://radish-genome.org) using Hisat2 (v2.0.5) [24]. The reads numbers mapped to each gene was counted by feature Counts v1.5.0-p3. FPKM (fragments per kilobase of exon per million mapped fragments) method was applied to count the gene expression levels. The DEGs were determined with an padj <  = 0.05 and |log2(foldchange)|> = 1 using R DESeq2 package. GO pathway enrichment analysis of DEGs were performed by the cluster Profiler R package (3.8.1) [25]. The P value threshold (P ≤ 0.05) was regarded as significantly enriched GO terms. Genome Analysis Toolkit (GATK, version v3.8) was used to call SNPs and InDels. SNPs and InDels were filtered with parameters (quality scores (QUAL) >  = 20 and quality by depth (QD) >  = 4). SnpEff (4.3q) software was implemented to annotate SNPs and InDels.

### Validation of InDel markers polymorphism

The length of primer pairs of InDels were varied from 18 to 24 bp, the predicted product sizes were ranged from 100 to 200 bp by Primer Premier 5.0 program. Polymerase chain reaction (PCR) reagents and amplification conditions were implemented as described previously by Luo et al. [3]. The PCR products were separated on 8% polyacrylamide electrophoresis (PAGE) gel. All validated primers of InDel markers were shown in Table S3.

### Genetic diversity, population structure and phylogenetic analysis

Popgene 32 program was used to determine values of the expected homozygosty and expected heterozygosity. PowerMarker version 3.2 was employed to calculate the number of alleles (Na), major allele frequency (MAF), and polymorphism information content (PIC) [26]. InDel markers were divided as three types: highly informative with PIC > 0.5), moderately informative with 0.25 < PIC < 0.5 and slightly informative with PIC < 0.25. Genetic structure analysis of radish accessions was conducted by the Structure 2.3.4 program [4]. The Bayesian model-based clustering was used to distribute individuals to groups with a predetermined number (K), which could be minimized Hardy–Weinberg and linkage disequilibrium within each group. The number of K was set from 1 to 10 with ten independent runs and 10,000 iterations were performed for estimation after a 10,000 iterations burn-in period. The subgroups and best K was estimated according to previous study [27]. Phylogenetic analysis and the dendrogram was carried out in previous studies [3].

### qRT-PCR analysis

Total RNA of eight flowing point in times, including vegetative growth, five vernalization times (5d, 10d,15d, 20 d, 25 d, 30d) and first flowering time were extracted and the cDNA of each sample was synthesized using the SuperScript III First-Strand Synthesis System (Invitrogen). The qRT-PCR experiment were performed as described previously [3]. The radish *Actin* were used as internal controls. The double delta CT (2^-ΔΔCt^) method was carried out to calculate relative transcription levels. Three technical replicates were conducted in each sample. The primer sequences were listed in Table S2.

## Results

### Genome resequencing

To development of molecular markers in radish, two radish cultivars, ‘YZH’ and ‘XHT’ have significant differences in flower time was used to genome resequencing. A total of 12.0 Gb and 12.6 Gb base pairs (bp) were generated in ‘YZH’ and ‘XHT’, respectively. After filtering the adapter sequences and low-quality reads, 79,949,206 and 84,185,286 clean reads in ‘YZH’ and ‘XHT’ were obtained (Table S4), of which 92.14% and 90.61% clean reads had a coverage depth of 26 and 23.7 of the radish reference genome in ‘YZH’ and ‘XHT’, respectively [24]. In total, 366,679 SNP and 97,973 InDel were identified in two cultivars (Additional file 3 and 4). The average frequency of SNP and InDel in the radish genome were calculated to be 1 SNP/1.22 kb and 1 InDel/4.6 kb. Most 39.7% (145,691) of the SNPs were located in intergenic regions, and 23.72% (86,973) were located in exonic sequence. The length distribution of InDels were ranged from 1 to 21 bp. The InDels length with 1 to 2 bp were the two most abundant type, accounting for 66.98% (65,632) of the total InDels (Fig. S1). Nine chromosomes of radish have different density, the highest number of InDels (21,734) were existed on chromosome 5, while the lowest number were displayed on chromosome 7 (6,416).

### Transcriptome sequencing and DEG analysis

**Fig. 1:**
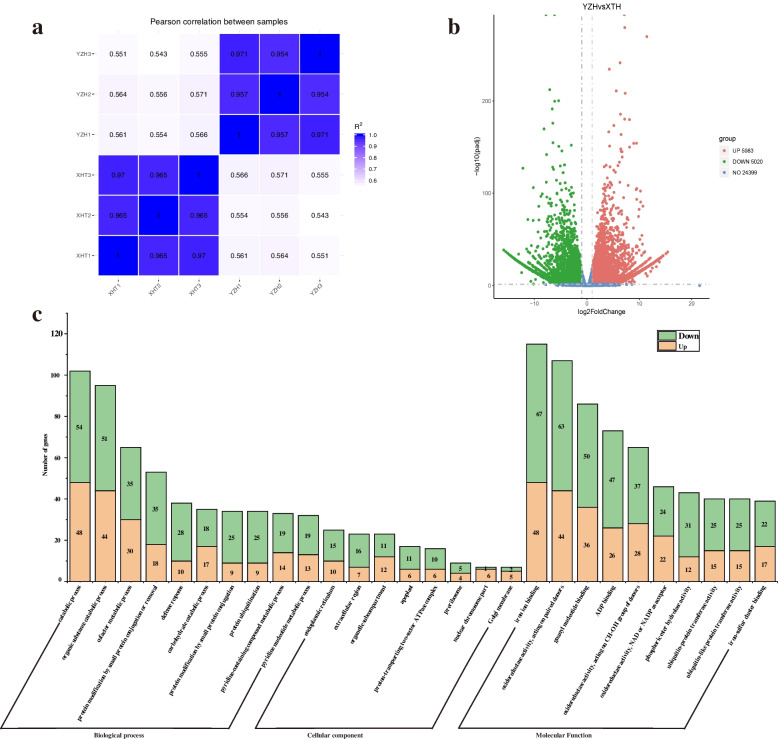
Differentially expressed genes (DEGs) and GO enrichment analysis. **a** Pearson correlation coefficient with three replicates in two cultivars. **b** Volcano plot showed the DGEs identified in two cultivars. **c** Enriched GO terms of the all DGE identified in two cultivars

To gain insights into gene expression changes and development of molecular markers in transcriptional level, a total of 6 cDNA libraries were sequenced in ‘YZH’ and ‘XHT’. In total, 29.87 Gb and 21.29 Gb clean reads were obtained (Table S7), 85.93% and 86.74% of the reads were mapped to the radish reference genome, of which 78.67% and 79.06% were uniquely mapped in ‘YZH’ and ‘XHT’, respectively. The Pearson correlation coefficient between the three replicates of each sample displayed higher values (> 0.95) (Fig. 1a). The principal component analysis (PCA) result indicated that PC1 and PC2 explained 90.83% of the total variation (Fig. S2).

To explore genes associated with flowering time, genes with |log2(FoldChange)|> = 1 and padj <  = 0.05 were defined as DEGs. In total, 11,003 DEGs were identified in between the two cultivars, of which 5,020 DEGs were upregulated and 5,983 DEGs were downregulated (Fig. 1b and Additional file 5). GO annotations reveals that all DEGs were assigned into 85 significantly enriched GO terms (Fig. 1c and Additional file 6). In the molecular function (43 terms), the major subcategories were iron ion binding, oxidoreductase activity, catalytic activity. For biological process (34 terms), catabolic process, organic substance catabolic process, cofactor metabolic process were the dominant terms. The ‘endoplasmic reticulum’ and ‘extracellular region’ terms were extraordinarily remarkable in the cellular component (8 terms). A total of 53,343 SNP were identified between two cultivars (Additional file 7), with an average frequency of 1 SNP/8.43 kb. The number of InDels with 1 to 2 bp was accounted for 72.23% (3,075) of the all InDels (4,257) (Fig. S1 and Additional file 8).

### Screening Validation and polymorphism of developed InDel markers

**Fig. 2:**
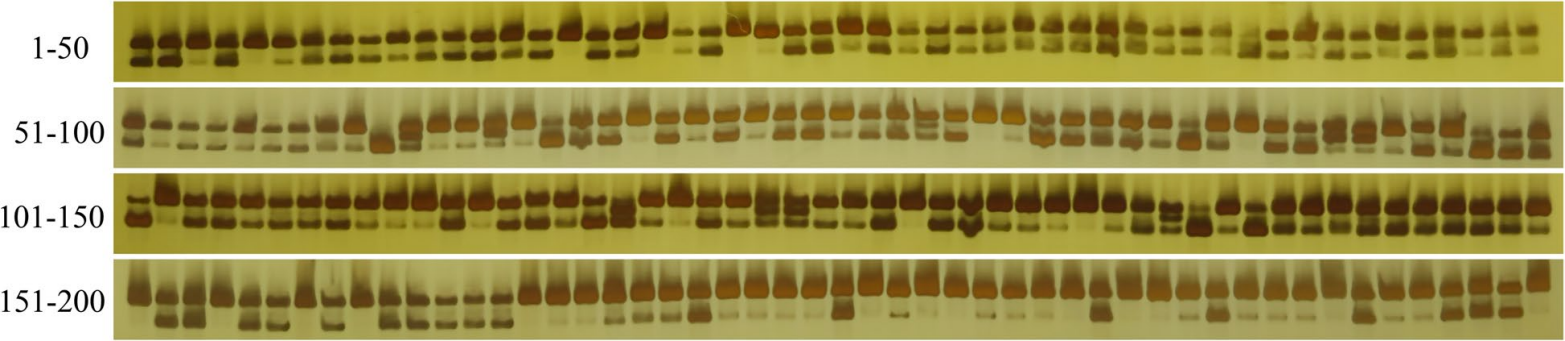
Polymorphism amplification results of the RsInDelR4-18 in 200 accessions

To validate the polymorphic InDel markers, 260 genomic and 40 transcriptomic InDels were randomly selected from genome resequencing and transcriptome sequencing of two cultivars, respectively. To facilitate the visualization of InDel markers on PAGE, the lengths of InDels greater than or equal to 3 were selected for PCR validation. The results showed that 85 genomic and 15 transcriptomic InDels were amplified successfully and exhibited polymorphisms in two cultivars, with an 32.7% an 37.5% marker polymorphism, respectively. The polymorphism amplification results of the RsInDelR4-18 primer in 200 radish cultivars were shown in Fig. 2. Consequently, the 100 polymorphic InDel markers were used for further analysis.

### Genetic diversity analysis and population structure

To assess the genetic diversity analysis of 200 Chinese radish accessions, 100 InDel primers were selected. The 100 polymorphic InDels markers generated 207 alleles among 200 Chinese radish germplasm. The number of alleles (Na) varied from 2 to 4, with an average of 2.07 alleles (Additional file 2). The major allele frequency (MAF) ranged from 0.48 to 0.93, with an average of 0.67. The expected homozygosity (HO) each InDel varied from 0.09 to 0.9, with an average of 0.62. The expected heterozygosity (HE) for each InDel ranged from 0.1 to 0.92, with an average of 0.38. The value of polymorphism information content (PIC) per locus ranged from 0.13 to 0.53, with an average of 0.33.

**Fig. 3:**
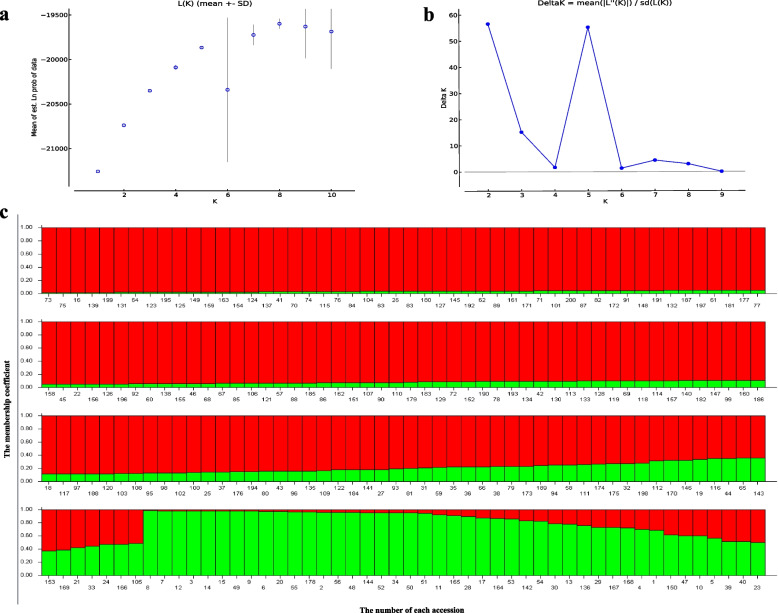
Population structure of the 200 radish accessions analyzed by STRUCTURE software. **a** Evaluateion of the optimal group number of △K. **b** L(K) (log probability of data) over ten runs for K ranging from 1 to 10. **c** The y-axis indicated the membership coefficient and the x-axis indicated the number of each accession

**Fig. 4:**
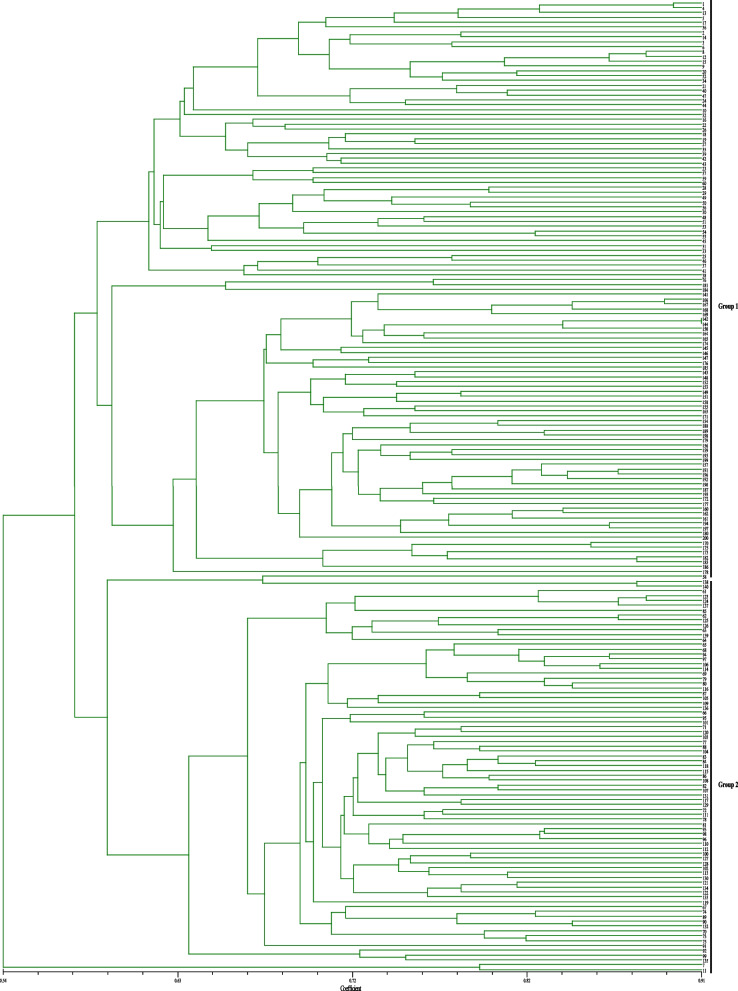
Dendrogram of cluster analysis for 200 radish accessions based on the 100 newly developed InDels

Population structure analysis was conducted based on the 200 radish accessions with 100 InDel markers using Structure 2.3.3 software. Delta K reached a maximum value at K = 2, indicating the 200 accessions could be divided into two groups (Fig. 3a and b). Apparently, six of seven accessions from Korea, one of two accessions from Japan, the majority of accessions with late flowering from northern China (Beijing, Shangdong, Heilongjiang) were clustered into the same group (Fig. 3c). The majority of accessions from southwest China (Guizhou, Yunnan, Sichuan) were clustered into same group. Cluster analysis implied that the 200 cultivars were divided into two groups with a genetic distance of 0.58 (Fig. 4). Group I contained 122 accessions, most varieties (58.4%) were derived from the northern China, seven accessions from Korea and two Japan were clustered into the same group. Group II contained 78 accessions, most of which (91.75%) were collected from the southwest China. The results of cluster and population structure analysis were basically consistent, but there were slight differences. These results demonstrated that the phylogenetic relationships among all accessions highly correlated with their geographical origins.

### Identification of homologous genes for flowering time

To identify homologs of flowering-time related gene (FTR) in radish, 306 FTR genes in Arabidopsis were downloaded from the Flowering Interactive Database (http://www.phytosystems.ulg.ac.be/florid/) and aligned to radish reference genome using BLASTN. Top hits with E-values ≤ 1^*E−20*^ and identity ≥ 80% were used to screen for the corresponding homologous genes. The 637 radish FTR genes were identified and divided into nine flowering-related pathways (Additional file 9). The largest number of FTR genes were involved in autonomous pathway (286). The smallest number of FTR genes were involved in sugar pathway (31) (Additional file 5: Table S8). The DEGs associated with the flowering pathway between two cultivars were screened with |log2 fold change|> 1 and FDR < 0.05. A total of 145 flowering time genes were identified, of which 74 were upregulated and 71 were downregulated (Additional file 10). The differentially expressed FTR genes were mainly associated with autonomous (50), photoperiod (39), and vernalization pathway (12). *Rs583930* and *Rs094390*, the homolog of *FT* plays key roles in flowering time integrator. In autonomous pathway, *UBIQUITIN CARRIER PROTEIN 1* (*UBC1*), *FLOWERING LOCUS Y* (*FY*), *AGAMOUS-LIKE 6* (*AGL6*), *FLOWERING LOCUS VE* (*FVE*), *RELATIVE OF EARLY FLOWERING 6* (*REF6*), *EMBRYONIC FLOWER 2* (*EMF2*) were detected. Several key genes in photoperiod pathway were also identified, such as *PSEUDORESPONSE REGULATORS 3* (*PRR3*), *PRR5*, *CYCLING DOF FACTOR 1* (*CDF1*), *CDF4*, *CDF5*, *CRY2*, *PHYCOCHROME A* (*PHYA*), *CALCIUM-DEPENDENT PROTEIN KINASES 6* (*CPK6*), *CPK33*, *PHYB*, *BBX19*, CONSTANS (CO), and *EARLY FLOWERING 4* (*ELF4*). Genes known to be involved in vernalization pathways were identified, such as *VERNALIZATION INSENSITIVE 3* (*VIN3*), *INDUCER OF CBF EXPRESSION 1* (*ICE1*), *FRIGIDA* (*FRI*), *AGL19*. *LEAFY* (*LFY*), *AGL25*, *AGL27*, *TCP18*, *APETALA 2* (*AP2*) involved in the flower development and meristem identity pathways were detected. Many vital genes in aging (*SPL15*), ambient temperature (*AGL31*, *PHYTOCHROME INTERACTING FACTOR 4* (*PIF4*), *AGL27*, hormone (*GA2ox1* and *GA2ox6*), *SUGAR SUCROSE TRANSPORTER 9* (*SUC9*), circadian clock (*CIRCADIAN CLOCK–ASSOCIATED 1*, *CCA1*), *PRR5* and flowering time integrator genes (*SUPPRESSOR OF OVEREXPRESSION OF CONSTANS 1*, *SOC1*) and FT were also detected.

Many studies reported that SNPs and InDels within or near the coding sequences (CDS) were significantly associated with important agronomic traits in crops. To identify of genetic variation in flower-related genes, SNP and InDel markers located in flower-related DGEs were detected. A total of 150 transcriptomic SNPs and 9 transcriptomic InDels within 30 and 6 differentially expressed FTR genes were obtained, respectively (Additional file 11). A total of 12 differentially expressed FTR genes with at least 2 SNPs were identified, such as *PHYA*, *TEM1*, *VIN3*, *PIF5*, *ADG1*, *FVE, ELF3*. A total of 3 differentially expressed FTR genes with at least 2 InDels were identified, including *ELF3, VIL2, TPS1*. These results provided valuable information on the explaining significant difference in flower time between ‘YZH’ and ‘XHT’.

### qRT-PCR validation

**Fig. 5:**
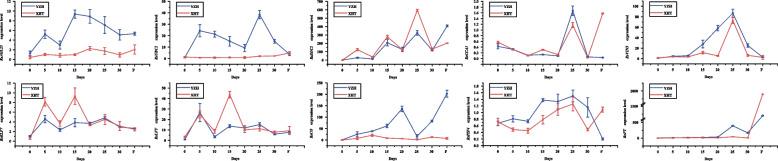
RT-qPCR validation of DEGs related to the flowering pathway in two cultivars. Each bar shows the mean ± SE of three replicate

To assess the expression pattern of DEGs identified from RNA-Seq, ten candidate DEGs involved in the flowering pathway were selected for qRT-PCR analysis in leaves of ‘YZH’ and ‘XHT’ (Fig. 5). The expression of five DEGs ( *RsAGL25*, *RsVIN3*, *RsUBC1*, *CO* and *RsPIF4*) were highly expressed in ‘YZH’ during eight times. *RsSOC1, RsCCA1, RsVIN3* were highly expressed in 30 d of vernalization. *RsELF7*, *RsLFY, RsVIN3* were highly expressed in 15 d of vernalization in ‘XHT’. *RsFT* were highly expressed in the first flowering time. These results revealed that these DEGs might involve in the phase transition from vegetative stage to bolting and flowering in radish.

## Discussion

### Identification of SNP and InDel markers in radish

SNP and InDel markers were the most abundant variations in the plant genomes, which had been widely utilized in genetic diversity analysis, gene mapping, genome wide association mapping and marker-assisted selection breeding [5, 28]. With the release of genome sequences information, providing important data reference for the resequencing of different radish varieties [21]. Although a large number of InDel markers have been extensively detected in radish, the number of InDel markers is still insufficient. In this study, 366,679 genomic SNPs and 97,973 genomic InDels were identified in ‘YZH’ and ‘XHT’, with an average frequency of 1 SNP/1.22 kb and 1 InDel/4.6 kb. The frequency of genomic SNP was significantly lower than previously described in radish (average occurrence of 3.9/kb) [21]. The frequency of genomic SNP and InDel in the radish were remarkably different with other plant species, including in *Arabidopsis* [29], *Brassica rapa* [30], tea plant [4]. The distinct filtering criteria and the different genetic structure among different plant species were likely to cause significant differences in SNP and InDel density [4]. In a previous study, 22,412 SNPs and 9,436 InDels were identified in three radish transcriptome [3]. In this study, 53,343 SNPs and 4,257 InDels were identified from transcriptomes sequencing data in two cultivars. The large discrepancy in SNPs and InDels number may be caused by different materials and SNP calling strategies. The short InDels (1–2 bp) were most prevalent types in the radish genome and transcriptome, which was coincided with previously studies in tea plant [4] and sesame [28]. Previous studies indicated that the InDel within the *RsRf3* locus played crucial roles in involving transition to fertility in cytoplasmic male sterility radish [31]. The SNP and InDel in *RsTT8* promoter were successfully distinguished between red and white-fleshed radish [32]. These studies demonstrated that SNP and InDel markers within functional genes were involved in important agronomic traits of radish. The newly identified SNP and InDel markers could provide abundant data information for the genetics and functional genomics research in radish.

### Development of InDel markers and application of germplasm resources

PCR based InDels with the advantages of co-dominant, inexpensive, and highly polymorphic were favored by more and more researchers in the field of gel based genotyping technology [33]. In this study, 85 genomic and 15 transcriptomic polymorphic InDels with an 32.7% and 37.5% marker polymorphism were validated by PCR based results, respectively, which was lower than previously reported results with 70% marker polymorphism [3]. The mean PIC value of InDels was 0.33 by genotyping of the 200 accessions, were lower than the PIC of the radish SSR markers, which can be explained that most InDels are single-locus, while SSRs are multi-locus [34, 35]. The alleles of single-locus markers can be positioned in the particular genomic loci, which was convenient to rapidly carry out genetic and breeding studies [36].

The genetic structure analysis of different genotypes is beneficial to develop varieties with a wide range of genetic backgrounds [7]. In the present study, 200 radish accessions were partitioned into two groups by population structure analysis. Apparently, the radish cultivars from northern China were clustered together, the radish cultivars from southwest China were clustered together. UPGMA cluster analysis revealed the 200 accessions were also clustered into two major groups. Previous studies indicated that flowering time are increased with latitude of origin [37]. It is important to introduce high latitude late flowering genes into radish. These results indicated that the InDel markers developed in this study was an important marker for genetic diversity analysis in radish.

### SNP and InDel markers within differentially expressed FTR genes

It has been widely accepted that the transition from vegetative to reproductive growth played important roles in the life cycle of an angiosperm plant [38]. The leaf tissues of early and late flowering time cultivars at the vegetative stage was conducted to perform transcriptome sequencing. A total of 145 flowering-time related DGEs were detected in radish by performing BLASTN analysis. Many studies have indicated that the InDel marker played crucial roles in the regulation of flowering time in radish [11, 39]. Vernalization promotes flowering in the late-flowering in plant. *FLC* encoding the MADS-box protein was a central floral repressor in *Arabidopsis* [40]. Previous studies found that the 1627-bp insertion in the first intron of *RsFLC2* gene in “Ninengo” plants, resulting in late-bolting [11]. One 9-bp deletion and two insertions (8 and 9 bp) were identified in the intron and promoter of *RsFLC3* gene [39]. In this study, the *RsFLC* genes were expressed in two cultivars, but no differentially expressed, illustrating *RsFLC* genes were no difference in vegetative phase for different cultivars. In wheat, *VRN1* was upstream of *FT* and induced the upregulation of *FT* expression under LD conditions [41]. In radish, a 647-bp insertion in promoter region of *RsVRN1* gene leaded the late-bolting phenotype in NAU-LB. The florigen *FT* played distinctive roles in regulation of the transition to reproductive development and flowering in plant [42]. The flower meristem-identity gene *LFY* played important roles in regulating *Arabidopsis* floral development [43]. Previous studies found that a 16-bp deletion and 18-bp deletion were detected in the intron of *FT* and *LFY* gene in radish [11]. In this study, the *RsFT* and *RsLFY* gene were differentially expressed in two cultivars by transcriptome sequencing and confirmed by qRT-PCR analysis.

In this study, SNP and InDel markers within differentially expressed FTR genes were identified, such as eleven SNPs and two InDels in *RsELF3* gene, ten SNPs in *RsTEM1* gene, five SNPs in *RsVIN3* gene, two SNPs and two InDels in *RsCDF5* gene, one SNPs in *RsFVE* gene, one SNPs in *RsEMF2* gene, one SNPs in *RsAGL6* gene, one SNPs in *RsCPK33* gene. *ELF3* gene regulated vegetative photomorphogenesis and the photoperiodic induction in Arabidopsis flower [44]. *TEM1* combinatorially interacted with *FT* rerepressed the floral transition in *Arabidopsis* [45]. The level of *VIN3* expression was associated with the duration of cold exposure and the degree of *FLC* repression in *Arabidopsis* [46]. Previous studies observed the *CDF5* protein delayed flowering through directly repressing *FT* transcription [47]. *FVE*, a component of the autonomous pathway involved in a protein complex repressed *FLC* expression [48]. Loss of function mutations of the *EMF2* genes leaded to early flower in *Arabidopsis*, confirming the important role of *EMF2* in phase transitions by restraining reproductive development [49]. *AGL6* enhanced *FT* expression in the *flc-3* background and the expression of *FLC* was downregulated in *agl6-1D* mutant [50]. *CPK33* was an important component of the florigen complex formation by *FD* phosphorylation [51]. These results could provide the marker data for the research of genes involve in bolting and flowering in radish.

## Conclusion

In this study, a large amount of genomic, transcriptomic SNPs and InDels were identified from genome and transcriptome sequencing between ‘YZH’ and ‘XHT’, respectively. A total of 100 novel InDel markers were developed and applied to genotype 200 radish accessions. Population structure and phylogenetic relationship revealed that the radish cultivars from northern China were clustered together and the southwest China cultivars were clustered together. RNA-Seq analysis demonstrated that 145 flowering time-related DGEs were detected, most of which were involved in flowering time integrator, circadian clock/photoperiod autonomous, and vernalization pathways. The transcriptomic SNPs and InDels within differentially expressed FTR genes were detected. These findings provide insights into the development and application of molecular markers for genetic diversity analysis, and provide the information of SNPs and InDels within flowering time-related DGEs in radish.

### Supplementary Information

**Additional file 1: Fig S1.**The length of genomic InDel markers between two radish cultivars.**Fig S2.**Principal component analysis of the all samples base on the FKPM vales of all transcripts.**Fig. S3.**The length of transcriptomic InDel markers between two radish cultivars.**Fig S4.** The full-length gels of the RsInDelR4-18 in 200 accessions. **Table S1.** Radish materials used in this study.**Table S2.** Primer information for qRT-PCR. **Table S4.** Summary of genome resequencing dada in two radish cultivars. **Table S7.** Summary of transcriptome dada in two radish cultivars. 

**Additional file 2: Supplement Table 3. **The primers of InDels used in this study and genetic diversity analysis data.

**Additional file 3: Supplement Table S5. **The information of SNPs between two radish cultivars in resequencing data.

**Additional file 4: Supplement Table S6. **The information of InDels between two radish cultivars in resequencing data.

**Additional file 5: Supplement Table S8. **The information of detected DEGs between two radish cultivars.

**Additional file 6: Supplement Table S9. **The enriched GO terms of DEGs between two radish cultivars.

**Additioanl file 7: Supplement Table S10. **The information of SNPs between two radish cultivars in transcriptome data.

**Additional file 8: Supplement Table S11. **The information of InDels between two radish cultivars in transcriptome data.

**Additional file 9: Supplement Table S12. ** Flowering time-related (FTR) genes in radish identified using Arabidopsis FTR genes as queries by BLASTN analysis.

**Additional file 10: Supplement Table S13. ** Identification of differentially expressed FTR genes in between two radish cultivars.

**Additional file 11: Supplement Table S14. ** The information of SNPs and InDels detected in differentially expressed FTR genes.

## Data Availability

Raw sequencing reads of RNA-seq in this paper have been deposited in the NCBI Sequence Read Archive (SRA) under BioProject accession number PRJNA874186.

## Abbreviations

MAS: Marker-assisted selection
RFLPs: Restriction fragment length polymorphisms
RAPDs: Random amplified polymorphic DNA
AFLPs: Amplified fragment length polymorphisms
SSRs: Simple sequence repeats
InDels: Insertion/Deletions
SNPs: Single-nucleotide polymorphisms
QTL: Quantitative trait locus
NGS: Next-generation sequencing
FLC: FLOWERING LOCUS C
FT: FLOWERING LOCUS T
FTR: Flowering-time related gene
DEGs: Differential expressed genes
PCR: Polymerase chain reaction
Na: Number of alleles
PIC: Polymorphism information content
PAGE: Polyacrylamide electrophoresis
MAF: Major allele frequency
PCA: Principal Component analysis
bp: Base pairs
HE: Expected heterozygosity
HO: Expected homozygosty
UBC1: UBIQUITIN CARRIER PROTEIN 1
FY: FLOWERING LOCUS Y
AGL6: AGAMOUS-LIKE 6
FVE: FLOWERING LOCUS VE
REF6: RELATIVE OF EARLY FLOWERING 6
EMF2: EMBRYONIC FLOWER 2
PRR3: PSEUDORESPONSE REGULATORS 3
CDF1: CYCLING DOF FACTOR 1
PHYA: PHYTOCHROME A
CPK6: Calcium-dependent protein kinases 6
CO: CONSTANS
VIN3: VERNALIZATION INSENSITIVE 3
ICE1: INDUCER OF CBF EXPRESSION 1
FRI: FRIGIDA
LFY: LEAFY
AP2: APETALA 2
PIF4: PHYTOCHROME INTERACTING FACTOR4
CCA1: CIRCADIAN CLOCK–ASSOCIATED1
SOC1: SUPPRESSOR OF OVEREXPRESSION OF CONSTANS 1
CDS: Coding sequences
LD: Long-day
FT: FLOWERING LOCUS T
