# Supplementary material for: Development of SNP and InDel markers by genome resequencing and transcriptome sequencing in radish (Raphanus sativus L.)
Source: BMC Genomics. 2023 Aug 8;24:445. doi: 10.1186/s12864-023-09528-6 (PMC10408230; doi:10.1186/s12864-023-09528-6)
Supplement: Supplementary file 1 — Additional file 1: Fig S1.The length of genomic InDel markers between two radish cultivars.Fig S2.Principal component analysis of the all samples base on the FKPM vales of all transcripts.Fig. S3.The length of transcriptomic InDel markers between two radish cultivars.Fig S4. The full-length gels of the RsInDelR4-18 in 200 accessions. Table S1. Radish materials used in this study.Table S2. Primer information for qRT-PCR. Table S4. Summary of genome resequencing dada in two radish cultivars. Table S7. Summary of transcriptome dada in two radish cultivars. [file 12864_2023_9528_MOESM1_ESM.docx]

**
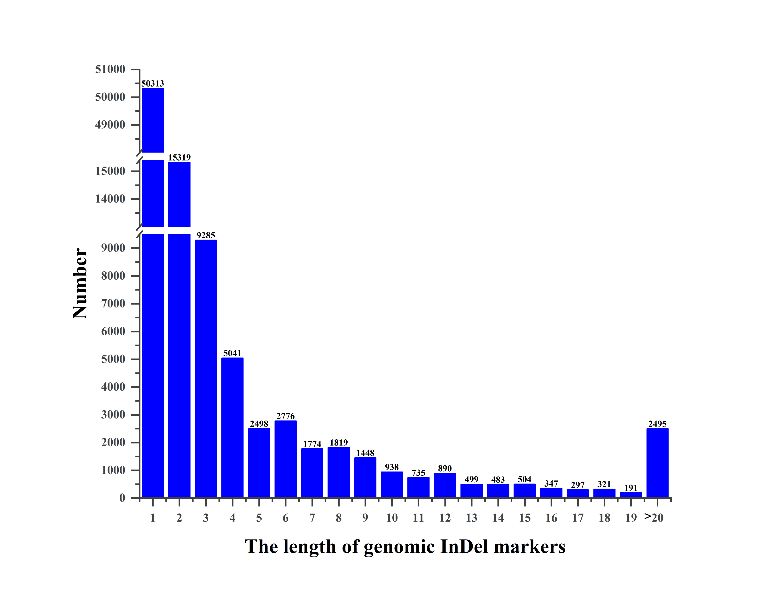
**

**Fig. S1.** The length of genomic InDel markers between two radish cultivars.

**
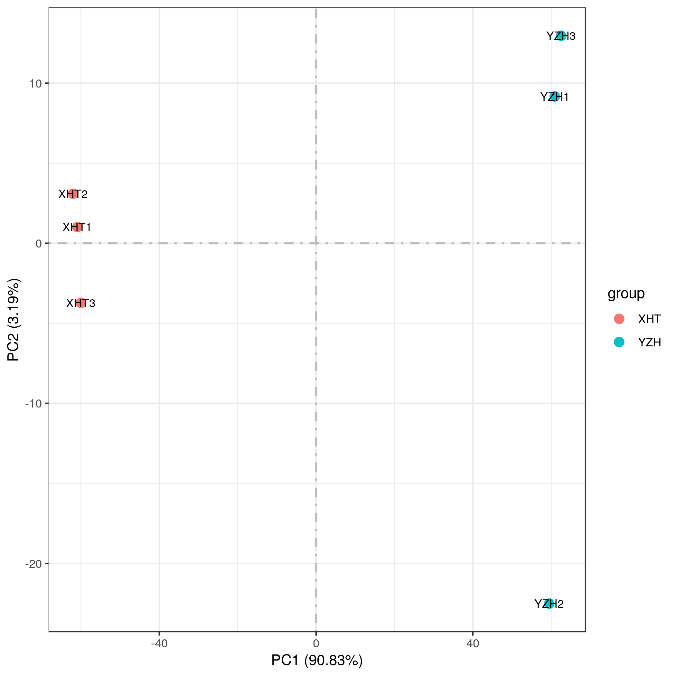
**

**Fig. S2.** Principal component analysis of the all samples base on the FKPM vales of all transcripts.

**
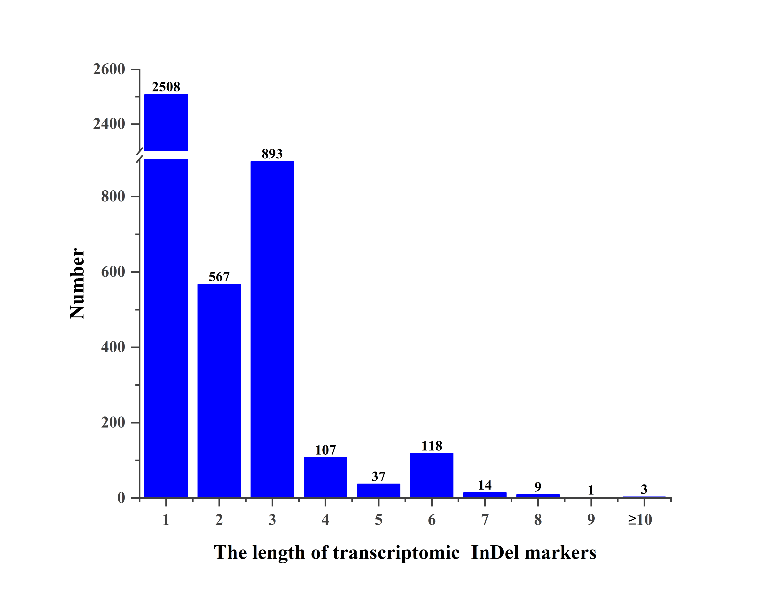
**

**Fig. S3.** The length of transcriptomic InDel markers between two radish cultivars.


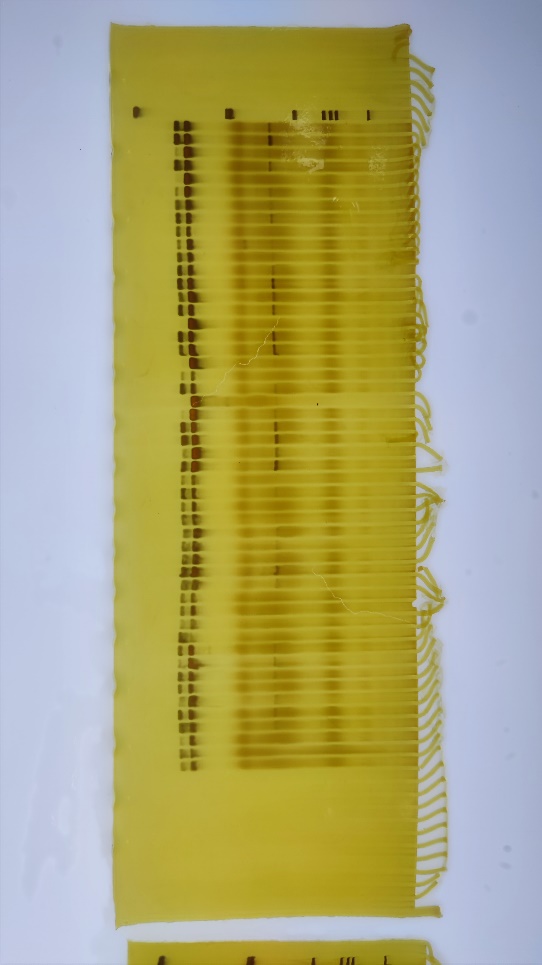


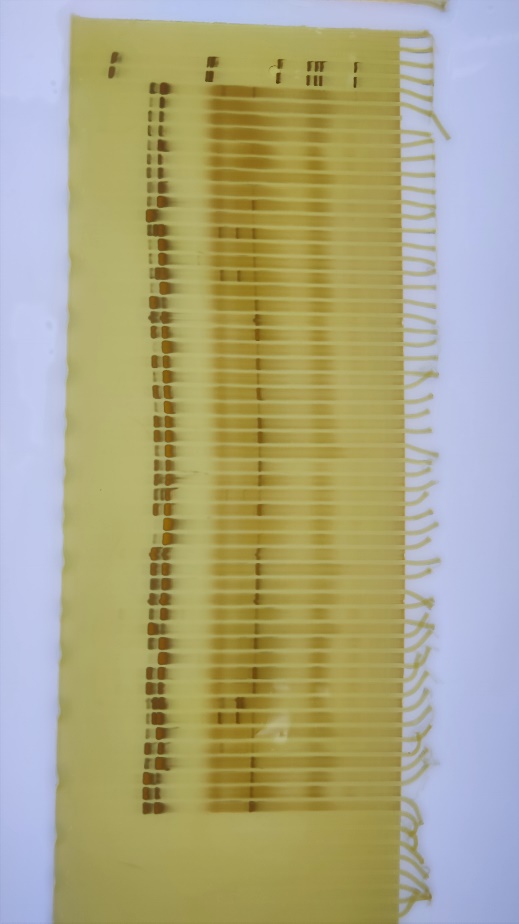


1-50


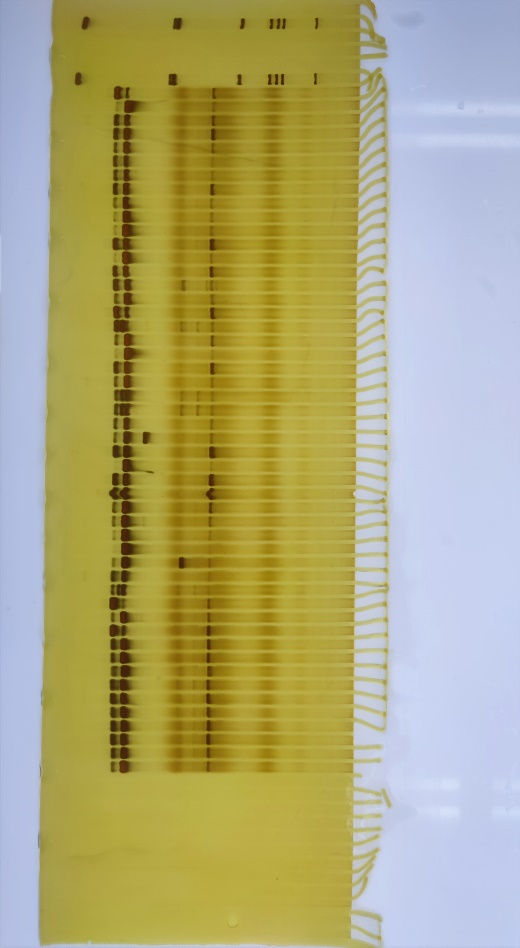


51-100


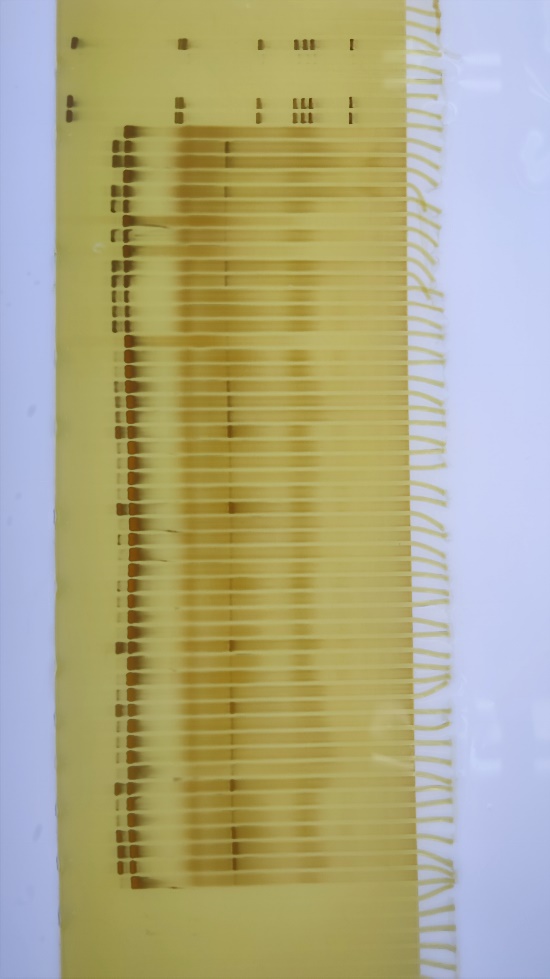


100-150

151-200

**Fig. S4.** The full-length gels of the RsInDelR4-18 in 200 accessions.

**Table S1.** Radish materials used in this study.

| Code | Name | Origin | [region](javascript:;) | flowering time | Code | Name | Origin | [region](javascript:;) | flowering time |
| --- | --- | --- | --- | --- | --- | --- | --- | --- | --- |
| 1 | Meilong123 | Beijing, China | northern China | 192 | 101 | Weining2 | Guizhou, China | southwest China | 178 |
| 2 | AA534 | Beijing, China | northern China | 191 | 102 | Weining3 | Guizhou, China | southwest China | 180 |
| 3 | AA173 | Beijing, China | northern China | 190 | 103 | Weining4 | Guizhou, China | southwest China | 167 |
| 4 | Jiutianjinyu | Hubei, China | northern China | 180 | 104 | Chengdumanshenhong | Sichuan, China | southwest China | 166 |
| 5 | Zhenbaiyu | Beijing, China | northern China | 180 | 105 | Ligenyihaoluobo | Yunnan, China | southwest China | 193 |
| 6 | Dizengbao4 | Beijing, China | northern China | 192 | 106 | Yun9 | Yunnan, China | southwest China | 183 |
| 7 | Yujiaolong | Beijing, China | northern China | 183 | 107 | Hongxinhongpiluobo | Sichuan, China | southwest China | 167 |
| 8 | Shengyu806 | Beijing, China | northern China | 183 | 108 | Weining5 | Guizhou, China | southwest China | 176 |
| 9 | Baigongbaoluo | Hubei, China | northern China | 183 | 109 | Zhengan1 | Guizhou, China | southwest China | 176 |
| 10 | JintianhouR2008 | Beijing, China | northern China | 191 | 110 | Zhengan2 | Guizhou, China | southwest China | 124 |
| 11 | Gaoyuanchunxue | Beijing, China | northern China | 176 | 111 | GD6 | Guizhou, China | southwest China | 171 |
| 12 | Jiutianhaoyu | Hubei, China |  | 191 | 112 | GD7 | Guizhou, China | southwest China | 174 |
| 13 | Dizengbao2 | Beijing, China | northern China | 191 | 113 | Weining6 | Guizhou, China | southwest China | 173 |
| 14 | AA1084 | Beijing, China | northern China | 191 | 114 | Xishui | Guizhou, China | southwest China | 179 |
| 15 | Junchuanyujie | Beijing, China | northern China | 186 | 115 | GD8 | Guizhou, China | southwest China | 164 |
| 16 | Hongmei | Beijing, China | northern China | 173 | 116 | GD9 | Guizhou, China | southwest China | 167 |
| 17 | Hanguodagen | Republic of Korea | Republic of Korea | 192 | 117 | Zhengan3 | Guizhou, China | southwest China | 166 |
| 18 | Banyemanshenhong | Sichuan, China | southwest China | 165 | 118 | Weining7 | Guizhou, China | southwest China | 172 |
| 19 | Manshenhong | Sichuan, China | southwest China | 165 | 119 | Daozhen1 | Guizhou, China | southwest China | 167 |
| 20 | Hanjinbaiyu | Republic of Korea | Republic of Korea | 192 | 120 | Daozhen2 | Guizhou, China | southwest China | 165 |
| 21 | Shangjiang9 | Beijing, China | northern China | 180 | 121 | Fenggang | Guizhou, China | southwest China | 174 |
| 22 | Banyechunbulao | Sichuan, China | southwest China | 161 | 122 | Daozhen3 | Guizhou, China | southwest China | 176 |
| 23 | Ribendagen | Japan | Japan | 176 | 123 | GD9 | Guizhou, China | southwest China | 186 |
| 24 | AA778 | Beijing, China | northern China | 173 | 124 | GD10 | Guizhou, China | southwest China | 186 |
| 25 | Degaodongshan | Shandong, China | eastern China | 180 | 125 | GD11 | Guizhou, China | southwest China | 186 |
| 26 | Shaguandaqingtou | Shandong, China | eastern China | 165 | 126 | GD12 | Guizhou, China | southwest China | 186 |
| 27 | Hongmandi | Sichuan, China | southwest China | 165 | 127 | Weining8 | Guizhou, China | southwest China | 174 |
| 28 | Hongshuailuobo | Heilongjiang, China | northeast China | 165 | 128 | Weining9 | Guizhou, China | southwest China | 180 |
| 29 | Hongpiyouzhimanshenhong | Sichuan, China | southwest China | 165 | 129 | Weining10 | Guizhou, China | southwest China | 176 |
| 30 | Shouerxueyu | Republic of Korea | Republic of Korea | 192 | 130 | GD13 | Guizhou, China | southwest China | 174 |
| 31 | Mutianhongfuerhao | Beijing, China | northern China | 173 | 131 | GD26 | Guizhou, China | southwest China | 174 |
| 32 | Hongpichengdumanshenhong | Sichuan, China | southwest China | 165 | 132 | Degaoqiuyu | Shandong, China | eastern China | 180 |
| 33 | Heiyuluobo | Beijing, China | northern China | 173 | 133 | GD14 | Guizhou, China | southwest China | 174 |
| 34 | Hanbaiyu | Republic of Korea | Republic of Korea | 192 | 134 | GD15 | Guizhou, China | southwest China | 174 |
| 35 | Sijimanshenhong | Sichuan, China | southwest China | 165 | 135 | GD16 | Guizhou, China | southwest China | 193 |
| 36 | GD25 | Guizhou, China | southwest China | 165 | 136 | GD17 | Guizhou, China | southwest China | 193 |
| 37 | Degaoqingquan | Shandong, China | eastern China | 180 | 137 | GDH4 | Guizhou, China | southwest China | 186 |
| 38 | Degaoboluomi | Shandong, China | eastern China | 180 | 138 | GDH2 | Guizhou, China | southwest China | 165 |
| 39 | Shengcuierhao | Shandong, China | eastern China | 184 | 139 | GDH3 | Guizhou, China | southwest China | 186 |
| 40 | Qingguan107 | Yunnan, China | southwest China | 173 | 140 | GDH1 | Guizhou, China | southwest China | 165 |
| 41 | AA1092 | Beijing, China | northern China | 180 | 141 | GD18 | Guizhou, China | southwest China | 173 |
| 42 | Guizu | Sichuan, China | southwest China | 186 | 142 | Hanguobaiyuchunluobo | Republic of Korea | Republic of Korea | 193 |
| 43 | Yanzhimanshenhong | Sichuan, China | southwest China | 165 | 143 | GD27 | Guizhou, China | southwest China | 176 |
| 44 | AA544 | Beijing, China | northern China | 179 | 144 | GD19 | Guizhou, China | southwest China | 178 |
| 45 | Shengcuiyihao | Shandong, China | eastern China | 184 | 145 | GD20 | Guizhou, China | southwest China | 167 |
| 46 | Yachunqingcui | Yunnan, China | southwest China | 180 | 146 | GD21 | Guizhou, China | southwest China | 170 |
| 47 | Shangjiang10 | Beijing, China | northern China | 180 | 147 | GD22 | Guizhou, China | southwest China | 174 |
| 48 | Jieliya709 | Beijing, China | northern China | 180 | 148 | Liushizaoluobo | Sichuan, China | southwest China | 174 |
| 49 | Hanxuebaiyu | Republic of Korea | Republic of Korea | 192 | 149 | Bantouhongyanzhiluobo | Sichuan, China | southwest China | 174 |
| 50 | Baiwang | Beijing, China | northern China | 180 | 150 | Yidianhongluobo | Zhejiang, China | eastern China | 173 |
| 51 | AA1054 | Beijing, China | northern China | 180 | 151 | Huasuzidaoxin | Shandong, China | eastern China | 176 |
| 52 | Yachun2 | Yunnan, China | southwest China | 192 | 152 | Yanzhihongluobo | Sichuan, China | southwest China | 170 |
| 53 | AA1053 | Beijing, China | northern China | 192 | 153 | Gailiang791 | Hebei, China | northern China | 167 |
| 54 | AA251 | Beijing, China | northern China | 180 | 154 | Beijingxinlimei | Beijing, China | northern China | 177 |
| 55 | AA980 | Beijing, China | northern China | 184 | 155 | Banxialuobo | Jiangsu, China | eastern China | 183 |
| 56 | Baishi | Beijing, China | northern China | 184 | 156 | Tianjingliwaiqing | Tianjin, China | northern China | 167 |
| 57 | XHT | Guizhou, China | southwest China | 186 | 157 | Beijinggaotangcui | Beijing, China | northern China | 180 |
| 58 | Yanzhihong1 | Guizhou, China | southwest China | 165 | 158 | Oulanbaiyu | Hebei, China | northern China | 186 |
| 59 | GDL | Guizhou, China | southwest China | 186 | 159 | Sijiqingluobo | Anhui, China | eastern China | 176 |
| 60 | DGE | Guizhou, China | southwest China | 131 | 160 | Tiancuishuiguolaobo | Hebei, China | northern China | 178 |
| 61 | GD3 | Guizhou, China | southwest China | 186 | 161 | Ribenfushiqing | Japan | Japan | 179 |
| 62 | GD4 | Guizhou, China | southwest China | 186 | 162 | Weixianqingshuiguoluobo | Shandong, China | eastern China | 178 |
| 63 | GD2 | Guizhou, China | southwest China | 183 | 163 | Chunbulao | Sichuan, China | southwest China | 174 |
| 64 | GD1 | Guizhou, China | southwest China | 184 | 164 | Jintianhou132 | Beijing, China | northern China | 193 |
| 65 | GD5 | Guizhou, China | southwest China | 182 | 165 | Jintianhou112 | Beijing, China | northern China | 193 |
| 66 | Hongfu | Sichuan, China | southwest China | 167 | 166 | Jintianhou948 | Beijing, China | northern China | 193 |
| 67 | Yanzhicuihongxinluobo | Chongqing,China | southwest China | 166 | 167 | Jintianhou906 | Beijing, China | northern China | 193 |
| 68 | Meihuachunbulaowanluobo | Guangxi, China | southern China | 180 | 168 | Jintianhou949 | Beijing, China | northern China | 193 |
| 69 | Xinxuanduanye13haokuaidaluobo | Guangxi, China | southern China | 121 | 169 | Jintianhou971 | Beijing, China | northern China | 193 |
| 70 | Tangjingluobo | Chongqing,China | southwest China | 173 | 170 | 20ridagen | Jiangsu, China | eastern China | 187 |
| 71 | Hongtaiyangluobo | Sichuan, China | southwest China | 167 | 171 | Jinghong30 | Beijing, China | northern China | 180 |
| 72 | Quanhongluobo | Sichuan, China | southwest China | 177 | 172 | Xiahongwrhao | Shandong, China | eastern China | 180 |
| 73 | Hanjintiancuiluobo | Sichuan, China | southwest China | 176 | 173 | Changfenghongyingtaoluobo | Jiangsu, China | eastern China | 186 |
| 74 | Baiyutangtianweiluobo | Sichuan, China | southwest China | 193 | 174 | Texuandaqingluobo | Hebei, China | northern China | 179 |
| 75 | Yuxingcuixue | Chongqing,China | southwest China | 173 | 175 | Shadingyangzhouyuanbailuobo | Jiangsu, China | eastern China | 183 |
| 76 | Xinlingmeiluobo | Sichuan, China | southwest China | 176 | 176 | Xiaoluobo | Jiangsu, China | eastern China | 176 |
| 77 | Yuxingyanzhihong | Chongqing, China | southwest China | 166 | 177 | 791guanluobo | Jiangsu, China | eastern China | 183 |
| 78 | Kunmingeryuehong | Yunnan, China | southwest China | 186 | 178 | Yulu218 | Jiangsu, China | eastern China | 180 |
| 79 | Pachixiaoluobo | Guangxi, China | southern China | 138 | 179 | Yangzhouyuanbai | Jiangsu, China | eastern China | 183 |
| 80 | Tejiduanyehuochekuaidaluobo | Guangxi, China | southern China | 121 | 180 | Jingcui | Hebei, China | northern China | 186 |
| 81 | Xinxuanhongluobo | Sichuan, China | southwest China | 167 | 181 | Mantanghong | Jiangsu, China | eastern China | 177 |
| 82 | Yuhongsanhao萝卜 | Chongqing,China | southwest China | 174 | 182 | Yingtaoluobo | Shandong, China | eastern China | 188 |
| 83 | Tiancuitouxinhong | Sichuan, China | southwest China | 167 | 183 | Hongxingyingtaoluobo | Gansu, China | northwest China | 184 |
| 84 | Junxiu | Sichuan, China | southwest China | 167 | 184 | GD23 | Guizhou, China | southwest China | 184 |
| 85 | Chuanhongyihao | Sichuan, China | southwest China | 165 | 185 | Tangbailuobo | Chongqing, China | southwest China | 173 |
| 86 | Yun8 | Yunnan, China | southwest China | 165 | 186 | Baitianshi | Beijing, China | northern China | 183 |
| 87 | Kunming | Yunnan, China | southwest China | 167 | 187 | Qingcuishuiguoluobo | Shandong, China | eastern China | 177 |
| 88 | Guanhongsanhao | Sichuan, China | southwest China | 164 | 188 | Fenglishuiguoluobo | Hunan，China | southern China | 181 |
| 89 | Yuxingheiye | Chongqing,China | southwest China | 186 | 189 | Zibaoluobo | Beijing, China | northern China | 192 |
| 90 | Ganshuiluobo | Sichuan, China | southwest China | 184 | 190 | Qinglongliuhaoluobo | Hebei, China | northern China | 181 |
| 91 | GD24 | Guizhou, China | southwest China | 172 | 191 | Qingguanzajiaoluobo | Heilongjiang, China | northeast China | 181 |
| 92 | Sijiyanzhihong | Sichuan, China | southwest China | 166 | 192 | Pingguocui | Shandong, China | eastern China | 173 |
| 93 | Yun5 | Yunnan, China | southwest China | 178 | 193 | Gaotangcuishuiguoluobo | Jilin，China | northeast China | 181 |
| 94 | Yun4 | Yunnan, China | southwest China | 180 | 194 | Jiaoshugailiangweixianluobo | Shandong, China | eastern China | 180 |
| 95 | Yun3 | Yunnan, China | southwest China | 174 | 195 | Tiancuishuiguoqing | Shandong, China | eastern China | 176 |
| 96 | Yun2 | Yunnan, China | southwest China | 173 | 196 | Qingcuiluobo | Heilongjiang, China | northeast China | 177 |
| 97 | Yun1 | Yunnan, China | southwest China | 180 | 197 | Weixianluobo | Shandong, China | eastern China | 181 |
| 98 | Yun6 | Yunnan, China | southwest China | 176 | 198 | Shouerzixingluobo | Republic of Korea | Republic of Korea | 193 |
| 99 | Yun7 | Yunnan, China | southwest China | 183 | 199 | Xinuotiancuiqing | Shandong, China | eastern China | 176 |
| 100 | Weining1 | Guizhou, China | southwest China | 174 | 200 | Qingtianyihao | Shandong, China | eastern China | 180 |

**Table S2.** Primer information for qRT-PCR.

| Gene ID | Gene model | Forward primer sequence | Reverse primer sequence |
| --- | --- | --- | --- |
| Rs178660 | RsUBC1 | ACTTGAATCTGGTAATGACTTAG | ACAACAACAACAACAATGATAG |
| Rs564610 | RsAGL25 | ATAACATAACAGCGAGACAA | GAAGATGATGAGAGCAACA |
| Rs054070 | RsVIN3 | GTGGTGATGATGAAGTAGAA | ATCCGAGCAAGTCATTATC |
| Rs311460 | RsLFY | CACCTATCTTCACAATCG | GCCTGCTCATATCTATCT |
| Rs398510 | RsCO | TAGAGGACCAAGAGTTCA | CATATAGCAGCATAAGTAATCAT |
| Rs263770 | RsPIF4 | AAGCCAAGTTCGTAGAGA | CATTGTCATTAGGTCTTCGTA |
| Rs365340 | RsCCA1 | GAGTAGTGATGATGTTGA | TGTGGATTGTTAGTAGTG |
| Rs210580 | RsSOC1 | GAAGAGGTGACGAAGAGA | TTGCTGTTGTTGGAAGAG |
| Rs094390 | RsFT | ACTGGTTGGTGACTGATA | CCTTGGACTCTCGTAAGA |
| Rs405380 | RsELF7 | GCTCTACATCTCTAACTTA | TCTTCCACTCTTATCATAG |
|  | RsACTIN | ATCAGGAAGGACTTGTACGGTAAC | GCTGAGGGAAGCAAGAATGGAACC |

**Table S4.** Summary of genome resequencing dada in two radish cultivars.

| Sample | Mapped reads | Total reads | Mapping rate(%) | Average depth(X) | Coverage at least 1X(%) | Coverage at least 4X(%) |
| --- | --- | --- | --- | --- | --- | --- |
| XHT | 77,566,742 | 84,185,286 | 92.14 | 26 | 79.16 | 75.15 |
| YZH | 72,440,232 | 79,949,206 | 90.61 | 23.74 | 80.15 | 75.55 |

**Table S7.** Summary of transcriptome dada in two radish cultivars.

| Sample | Total_reads | Total_map | Unique_map | Multi_map | Read1_map | Read2_map | Positive_map | Negative_map |
| --- | --- | --- | --- | --- | --- | --- | --- | --- |
| XHT1 | 42901466 | 37750627(87.99%) | 34364047(80.1%) | 3386580(7.89%) | 17202677(40.1%) | 17161370(40.0%) | 17192254(40.07%) | 17171793(40.03%) |
| XHT2 | 42586028 | 36817072(86.45%) | 33577355(78.85%) | 3239717(7.61%) | 16946662(39.79%) | 16630693(39.05%) | 16772881(39.39%) | 16804474(39.46%) |
| XHT3 | 42678722 | 36604338(85.77%) | 33390051(78.24%) | 3214287(7.53%) | 16868834(39.53%) | 16521217(38.71%) | 16676221(39.07%) | 16713830(39.16%) |
| YZH1 | 43358432 | 37254497(85.92%) | 33852866(78.08%) | 3401631(7.85%) | 17131437(39.51%) | 16721429(38.57%) | 16922783(39.03%) | 16930083(39.05%) |
| YZH2 | 43397142 | 37356308(86.08%) | 33950157(78.23%) | 3406151(7.85%) | 17152546(39.52%) | 16797611(38.71%) | 16966153(39.1%) | 16984004(39.14%) |
| YZH3 | 55214366 | 48399761(87.66%) | 44011347(79.71%) | 4388414(7.95%) | 22126561(40.07%) | 21884786(39.64%) | 22030398(39.9%) | 21980949(39.81%) |
